# Continuous spectroscopic monitoring of urinary catheter output: advancements and clinical implications

**DOI:** 10.1038/s41598-025-92802-2

**Published:** 2025-03-12

**Authors:** Sebastian Kuenert, Anastasia Meckler, Leonardo Poggi, Lukas Schipper, Thanusiah Selvamoorthy, Bernadette Hosters, Eva-Maria Huessler, Felix Nensa, René Hosch, Michael Fabian Berger, Mario Vincent Roser, Ramsi Siaj

**Affiliations:** 1 Department of Pediatric Surgery, University Hospital Essen, Essen, Germany; 2 Institute of Diagnostic and Interventional Radiology and Neuroradiology, University Hospital Essen, Essen, Germany; 3 Institute for Artificial Intelligence in Medicine (IKIM), University Hospital Essen, Essen, Germany; 4 Institute for Medical Informatics, Biometry and Epidemiology, University of Duisburg-Essen, Essen, Germany; 5 Department of Nursing Development and Nursing Research, University Hospital Essen, Essen, Germany; 6 Elixion Medical GmbH, Düsseldorf, Germany

**Keywords:** Urine diagnostics, Urinalysis, Spectroscopic monitoring, Electronic monitoring systems, Real-time analysis, AI in medicine, Biomarkers, Health care, Diagnosis, Mathematics and computing, Optical techniques, Optical spectroscopy

## Abstract

Urine diagnostics are crucial for identifying urological disorders and systemic diseases. Monitoring bladder catheters for urine output and early signs of urinary tract infections (UTIs) is essential but labor-intensive and prone to documentation errors. Recent advances in electronic monitoring and spectroscopy offer potential improvement in this process. This study introduces a novel smart sensor system for urinary catheters, enabling digital, continuous, automated real-time spectroscopic urine monitoring. A prototype consisting of a mini-spectrometer integrated with a custom lens array and hyperspectral illumination source was developed for urine analysis. It measures light intensity across 288 channels (340–850 nm) from multiple angles and exposure times, capturing detailed spectral data. Analyzing 401 urine samples from 168 patients, statistical models in R software were used to assess the dependency between spectral data and clinical laboratory values. The prototype accurately detected response variables like bilirubin, erythrocytes, pH, protein, specific gravity, and urobilinogen, with AUC values indicating good to very good discrimination. Response variables like glucose and nitrite, which do not absorb within the measured spectrum, showed minimal correlation. Our smart catheter system presents the potential for a significant advancement in urine monitoring, providing continuous, accurate analysis of parameters absorbing within the visible light spectrum.

**Supplementary Information:**

The online version contains supplementary material available at 10.1038/s41598-025-92802-2.

## Introduction

Urine diagnostics play a crucial role in clinical practice, enabling the prompt and sensitive identification of various pathologies, including urological disorders as well as systemic diseases affecting the kidneys^1^.

It is imperative to closely monitor bladder catheters for two primary reasons: first, to facilitate urine diagnostics and accurately assess output volume and second, to detect early signs of urinary tract infections or bleeding. Additionally, the diagnosis and monitoring of acute and chronic kidney diseases can be effectively managed in catheterized patients through observation of fluid balance and urine analysis.

In the hospital setting, the responsibility of catheter monitoring predominantly falls upon nursing staff. However, this practice is characteristically not automated and often done manually. Thus, it is time-consuming, resource-intensive, and prone to errors in documentation. Previous studies have reported a significant proportion of input and output records to be unreliable, with nearly 40% of records being inaccurate, indicating the need for more efficient monitoring methods^2,3^.

Significant advancements have been made in the field of electronic monitoring systems, which offer the potential to expedite the detection of Catheter-associated urinary tract infections (CAUTI)^4,5^. Additionally, spectroscopic techniques have shown promise in the realm of urine diagnostics, with studies successfully correlating urine spectra with clinically observable urine components^6,7^.

Building upon existing approaches and recognizing the limitations of current practices in a similar field, our research group conducted a pilot study focusing on the continuous monitoring of postoperative abdominal drain output. This study introduced an innovative integrated smart sensor system capable of real-time characterization and digitalization of drain output at the patient’s bedside^8^.

In this publication, we aim to extend this technology to urine catheters to show that the continuous spectroscopic monitoring of urine and its relevant diagnostic parameters holds significant benefits for both patients and healthcare institutions.

## Material and methods

### Prototype development

This study characterized urinary catheter output using a compact, affordable mini-spectrometer (Hamamatsu Photonics, CM12880MA), capable of rapid, non-obstructive measurements within the detection range of 340–850 nm. The spectrometer captured light intensity values from 0 to 65,000 (unitless) across 288 discrete detection channels, surpassing the human eye’s perception range of 360–830 nm as defined by the International Commission on Illumination^9^.

To facilitate future integration into a bedside urinary catheter system, a compact, energy-efficient, robust, and affordable light source capable of illuminating the entire detection range of the spectrometer was required. As no commercially available source met these specifications, a custom hyperspectral light source was developed. This source (illustrated in Supplementary Figure S1) combined three LEDs emitting in ultraviolet (UV), near-infrared (NIR), and full-spectrum (FS) ranges. The LEDs were mounted on a carrier PCB, and their light output was collected via a laser-cut light guide and directed to the sample chamber. When activated, all three LEDs operated simultaneously, producing hyperspectral illumination.

**Fig. 1:**
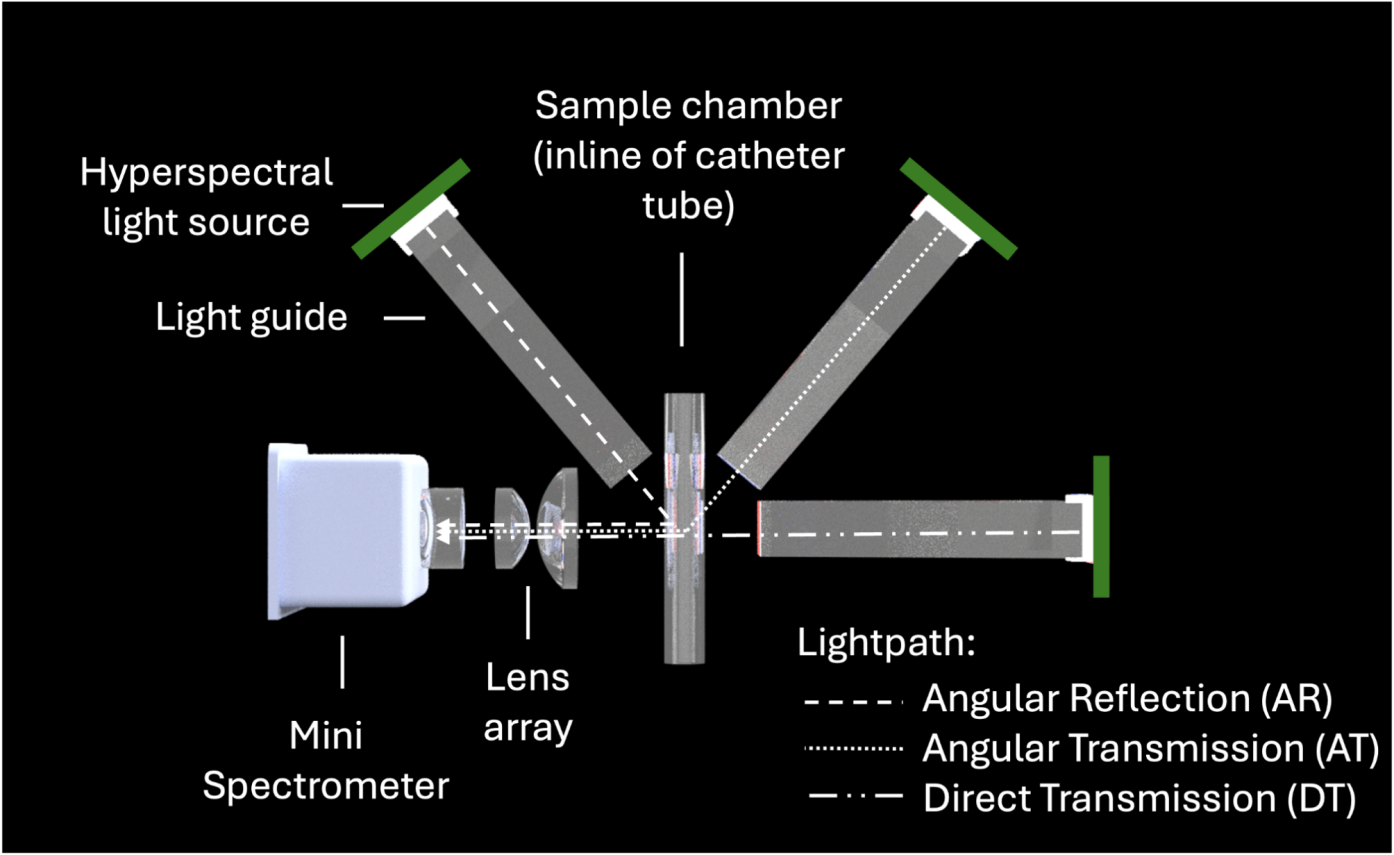
Schematic of the mini spectrometer and its key components. Light from the hyperspectral light sources is directed via the light guides to the sample chamber where it interacts with the sample. The resulting light output is captured by a lens array and focused on the spectrometer head. This setup utilizes three light sources placed at different angles to form three distinct light paths: direct transmission (DT), angular transmission (AT), and angular reflection (AR). This figure was rendered using Autodesk Fusion 360.

To assess the influence of different light paths on sample interaction, three configurations were evaluated: direct transmission (DT), angular transmission (AT), and angular reflection (AR). Optical simulations were performed using Ansys Zemax OpticStudio 2023 R2.02 (Supplementary Figure S2) to optimize the setup, including light source positioning, light guide geometry, and lens array configurations. Simulations prioritized minimizing light interaction with the glass walls of the sample chamber while maximizing light interaction with the fluid. The optimal setup, shown in Fig. 1, integrated the mini-spectrometer with a custom-designed lens array and three hyperspectral light sources, forming three distinct light paths: DT, AT and AR.

All materials in the optical path (light guide: Röhm, Plexiglas GS; sample chamber: Hilgenberg, borosilicate glass; lens array: Edmund Optics, UV quartz glass lenses) were selected based on their transmissivity within the spectrometer’s detection range.

Initially, the optical components were positioned using an evaluation platform constructed via Fused Deposition Modeling (FDM) 3D printing (Ultimaker S5) using polylactide (PLA). However, the lack of rigidity and low resolution of FDM printing led to unreliable positioning of the optical elements. To address this, an enhanced platform was developed using high-resolution Stereolithography (SLA) 3D printing (Formlabs Form 3B+) and rigid resin materials (Formlabs Tough Resin 1500). This improved platform ensured stable and precise positioning of the optical elements and incorporated mechanical isolation for critical components.

For data acquisition, six spectra were recorded for each fluid sample, corresponding to illumination from three distinct light paths (DT, AT and AR) and two exposure times (low and high). This approach was designed to optimize the signal-to-noise ratio while preventing oversaturation. To eliminate interference between light paths, spectra were collected separately, with only one hyperspectral light source activated at a time.

The spectral data were saved as comma-separated values (.csv) files, resulting in a cuboidal dataset comprising 3 light paths × 2 exposure times × 288 wavelength channels, producing 1,728 light intensity values influenced by the fluid’s biochemical properties. These data were collected for subsequent statistical analysis.

### Study population andethics committee

A total of 401 samples from 168 patients of the University Hospital Essen were collected for this study. Informed consent was obtained from adult patients as well as written consent from legal guardians as well as assent from minors. The study received ethical approval under reference number 21-10402-BO.

All methods were conducted in accordance with relevant guidelines and regulations, including the principles outlined in the Declaration of Helsinki.

Detailed information regarding the number of participants, age and gender distribution can be found online in Supplementary Figure S3.

### Urine parameters and sample collection

Table 1, under the column “Urine Marker” presents the urine parameters measured in this study along with the methodologies employed for their assessment. Parameters labeled quantitative were measured by the central laboratory of the University Hospital Essen using specific detection methods resulting in data points on the metric scale. Parameters labeled qualitative were measured using urine test strips, providing ordinally scaled data points.

Samples were either collected from catheter bags or passed directly into sample collection tubes by the patients. An overnight waiting period of at least 12 h was observed between sample collections for patients providing multiple samples. The number of samples collected per patient ranged from one to eleven, with a mean of 3.04 samples per patient and a standard deviation of 2.34. The variation in the number of samples was influenced by the duration of each patient’s stay on the ward.

Following collection, urine samples were immediately split into two fractions (“A” & ”B”), frozen at -80 °C, and stored until further processing.

### Measurements

Urine samples were measured in batches of 20 to 40 samples (“A”) after thawing at room temperature, utilizing the described prototype. For each measured sample, a corresponding frozen sample (“B”) was sent to the central laboratory at Essen University Hospital to obtain clinically validated values for the parameters as presented in Table 1.

Following each measurement, the urine sample was discarded, and the prototype tubing and measurement chamber were thoroughly flushed with sterile water to prevent cross-contamination between samples. After completing the batch measurements, the prototype underwent a flush with isopropyl alcohol followed by water to ensure the prevention of sample cross-contamination and bacterial growth.

### Sample overview

After sample analysis at the central laboratory, an overview of the ratio of pathological vs. healthy samples for each urine parameter was created (Table 1).

**Table 1:**
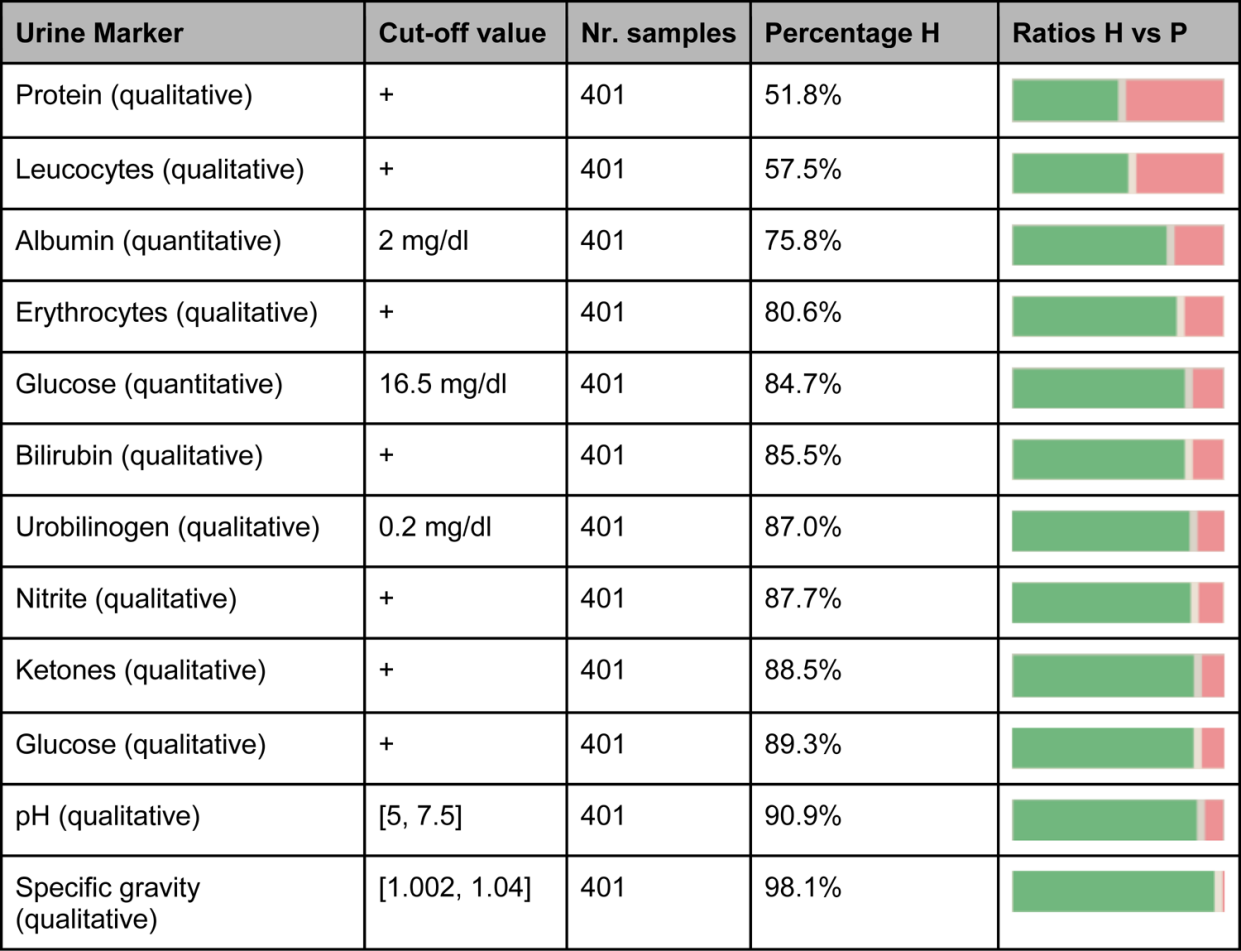
Overview of the urine markers considered in this study.

A marker is categorized as pathological if its value is less than or equal to the corresponding cut-off value, except for pH, where healthy samples are within the range of 5 to 7.5. The table also includes the percentage of healthy samples for each binarized marker, along with a visual representation of the distribution of pathological (“P”, red bars) and healthy samples (“H”, green bars).

Table 1 provides an overview of the measured urine parameters, detailing the methodology of data acquisition, the total number of obtained and measured samples, the respective cut-off values for healthy samples, and the distribution of pathological and healthy samples. The column labeled “Percentage H” presents the proportion of healthy samples within the entire sample set, where healthy samples constitute the predominant class across all parameters. A visual representation of this ratio is depicted in the “Ratio H vs. P” column, where green bars signify healthy samples and red bars indicate pathological samples.

The Cut-off value column specifies the cut-off values between healthy and pathological samples. For most samples, the range provided by the central laboratory of the University Hospital Essen was adopted. However, for urine parameters pH and specific gravity, no range was specified by the central laboratory. Accordingly, the range was determined by literature research with a range of urine pH of [5, 7.5]^10^ and a range of urine specific gravity of [1.002, 1.04]^11^.

Urine parameters were organized based on the proportion of healthy samples, revealing that parameters such as protein and leukocytes exhibited approximately equal proportions of healthy and pathological samples, with fewer pathological samples observed for all other parameters.

Given the low number of pathological samples observed for the pH parameter and its potential for straightforward statistical analysis, it was not categorized into a distinct healthy and pathological class. Instead, it was bifurcated into two parameters (pH acidic and pH basic) and analyzed according to the acidity/alkalinity of the sample, with samples exhibiting a pH of 7 being considered neutral in both parameters.

### Statistical analysis and programming

#### Spectra normalization

The correct calibration of the spectrometer is crucial for the acquisition of clean data. Nevertheless, artifacts caused by external events, such as noise contribution, and the physical properties of the sample (e.g., turbidity), can significantly affect the measured light intensities between different spectra^11^. When comparing different spectra, pronounced scattering in the intensity values becomes evident, creating a bias in the input data that can negatively impact the outcome of statistical analysis methods^12^. In the present work, correction for such phenomena was achieved by scaling the spectra using the Standard Normal Variate (SNV) method. The SNV method transforms a spectrum $s\to$into a new spectrum $\stackrel{\sim}{s}\to$ by mapping each datapoint ${s}_{i}\in s\to$ to a new datapoint $\stackrel{\sim}{{s}_{i}}\in\stackrel{\sim}{s}\to$using the following equation:

$$\stackrel{\sim}{{s}_{i}}=\frac{{s}_{i}-\mu(s\to)}{\sigma(s\to)}$$

where $\mu(s\to)$ and $\sigma(s\to)$are, respectively, mean and standard deviation of the original spectrum $s\to$. As a consequence, the transformed spectrum $\stackrel{\sim}{s}\to$ has zero mean and unit variance.

**Fig. 2:**
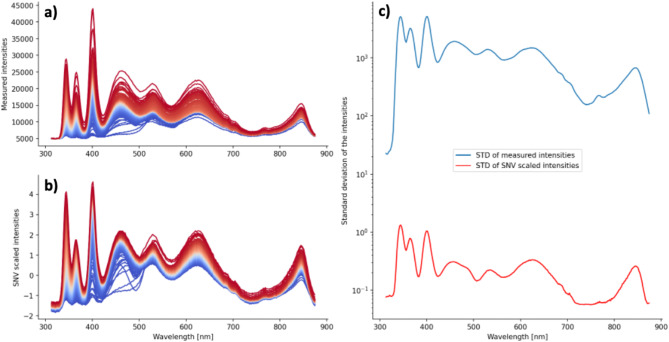
SNV correction on the spectral data measured in attenuated reflection with 320 ms exposure time (AR_320). (**a**) Original spectra. (**b**) Spectra after the SNV correction. (**c**) Semi-logarithmic plot of the standard deviation of the intensities before (blue line) and after (red line) the SNV correction.

The effect of the SNV method is shown in Fig. 2. In this example, all spectra measured in attenuated reflection with an exposure time of 320 ms (AR_320) are plotted together before (Fig. 2a) and after (Fig. 2b) the SNV correction. The impact of this scaling method on the variance of the data is depicted in Fig. 2c. For each wavelength, the standard deviation of the intensities of the spectra before (blue line) and after (red line) the SNV correction is shown in a semi-logarithmic plot. With average values of 1097.62 (before SNV) and 0.24 (after SNV), the standard deviation of the intensities at each wavelength decreased drastically by nearly 4 orders of magnitude. Similar results were obtained for all other spectrometer settings, defined by combination of light pathway and exposure time.

#### Correlation analysis

Next, a correlation analysis guided by literature about the known optical characteristics of the respective marker was performed to identify the wavelengths in the spectra that show the highest correlations with the measured laboratory parameters, narrowing down the range of wavelengths to the most relevant for further statistical modeling.

**Fig. 3:**
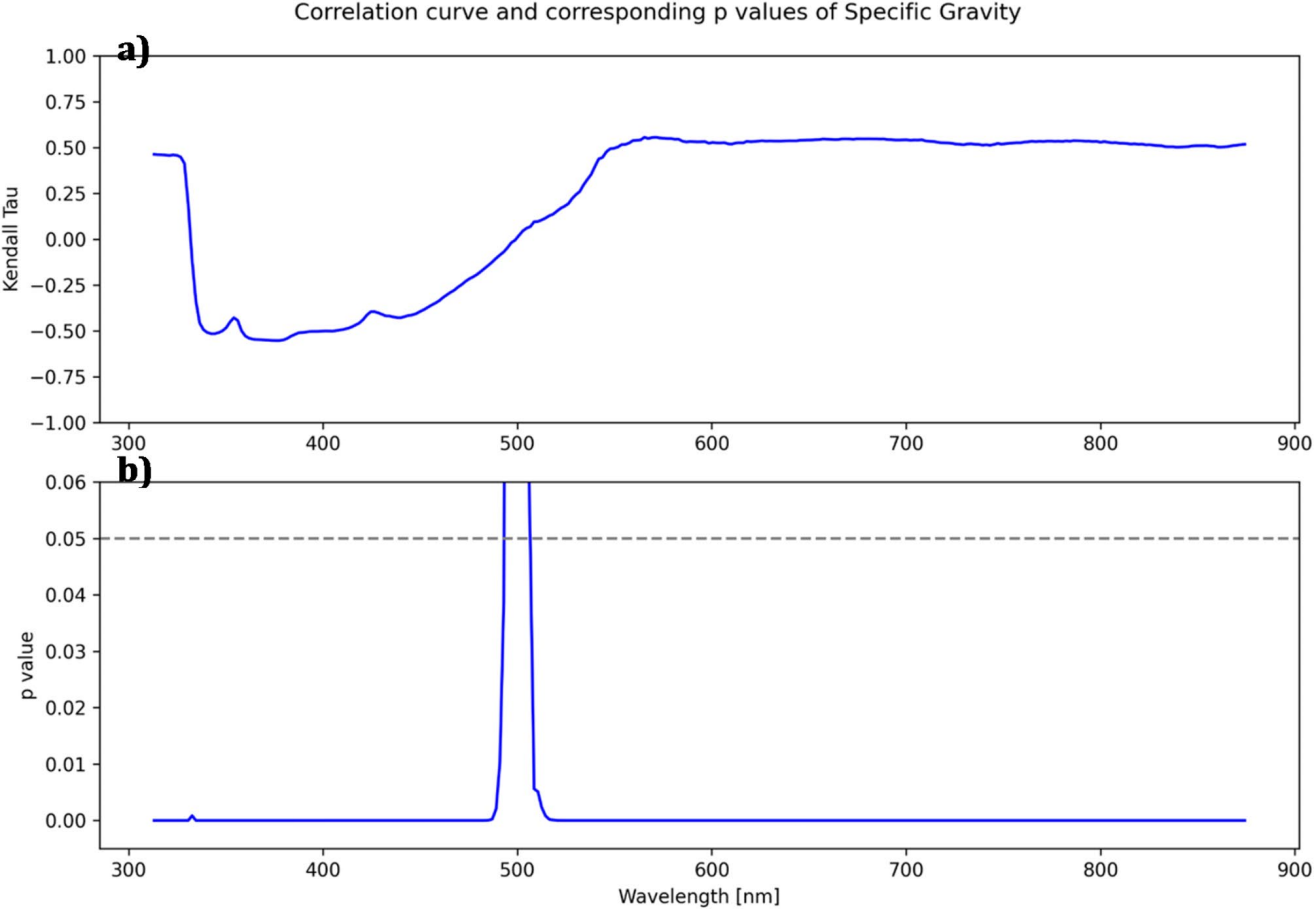
Correlation curve (**a**) and corresponding $p$-values (**b**) for the laboratory parameter specific gravity and spectrometer in direct transmission with 20 ms exposure time (DT_20).

The measured spectra for a specific spectrometer setting were arranged in an $nx288$ matrix $S$ where $n$ represents the number of samples. Each column vector ${s}_{i}\to$ of the matrix contained all the scaled intensities measured at the $i$-th wavelength. Similarly, for a specific laboratory parameter, a vector $l\to=({l}_{1},{l}_{2,\dots,}{l}_{n})$ was defined containing the measured laboratory value of each sample. A correlation coefficient and corresponding $p$-value, indicating whether the correlation coefficient is significantly different from zero, were computed between each column vector ${s}_{i}\to$ and the laboratory vector $l\to$. This process was repeated for each spectrometer setting and laboratory parameter. If the laboratory parameter was measured quantitatively, the Pearson’s correlation coefficient was computed; otherwise, Kendall’s ττ correlation coefficient was chosen for the correlation analysis. The calculated coefficients were wavelength-specific and were arranged into correlation curves. An example of such curves for the laboratory parameter “specific gravity” and spectrometer setting DT_20 is shown in Fig. 3a. The corresponding $p$-values in Fig. 3b provide a measure of the statistical significance of the computed correlations.

### Statistical modeling methodology for spectral data analysis and urine parameter classification

All analyses were performed using R version 4.4.1^13^ and RStudio^14^, utilizing the following R packages: glmtoolbox^15^, lme4^16^, MuMIn^17^, caret^18^, ConfusionTableR^19^ and ROCR^20^.

To ensure the reliability and quality of the dataset, specific data points were excluded from the analysis. Measurements taken at higher exposure times, which resulted in light intensities exceeding the spectrometer’s maximum capacity and causing detector oversaturation, were removed from the dataset across all three illumination angles. No additional data omissions were made intentionally.

The response variables were dichotomized based on the urine markers and their respective cut-off values outlined in Table 1. For qualitatively measured outcomes, generalized linear models (glm) with logit link function (logistic regression models) were estimated. Spectral data at various wavelengths were used as regressor variables. To account for patient-specific variability in the dataset, a logistic regression model (denoted as LRRE) incorporating a random intercept for each patient was estimated. This approach assumes that the relationship between predictors and outcomes is consistent across all patients, while the random intercept accounts for between-patient variability^21^. For comparison, a logistic regression model without random intercepts (LR) was also computed.

The classification performance of the models was evaluated using two key metrics: the Area Under the Curve (AUC) of the Receiver Operating Characteristic (ROC) curve and Balanced Accuracy (BAC). BAC is defined as the mean of sensitivity and specificity. To identify the most relevant regressors for model performance, a best subset selection procedure was performed by minimizing the Akaike Information Criterion (AIC).

The best subset selection and model estimation were carried out using the entire dataset (*n* = 401) to derive a global model. The AUC calculated for the global model represents its inner-sample performance, as the same dataset was used for both subset selection and model estimation. However, since inner-sample performance tends to be overly optimistic, an out-of-sample performance estimate was required to account for potential overfitting.

To estimate out-of-sample performance, a correction term was derived using a 5-fold cross-validation (CV) procedure. The dataset was divided into five subsamples of approximately equal size (80 or 81 observations each). In each fold, four subsamples were combined to form the training dataset (*n* = 320/321), while the remaining subsample (*n* = 80/81) was used as the test dataset. This process was repeated five times, ensuring that each subsample served as the test dataset once.

For each training dataset, best subset selection and model estimation were conducted, and the inner-sample performance of the resulting model was evaluated. The out-of-sample performance was then assessed by applying the model to the corresponding test dataset. The differences between inner- and out-of-sample performance were calculated for each of the five folds. The mean of these differences was used as the correction term. By subtracting this correction term from the inner-sample performance of the global model, an estimator for the global model’s out-of-sample performance was obtained.

## Results

### Correlation analysis

After performing correlation analysis as described above, independent variables were selected from the most promising wavelengths.

**Fig. 4:**
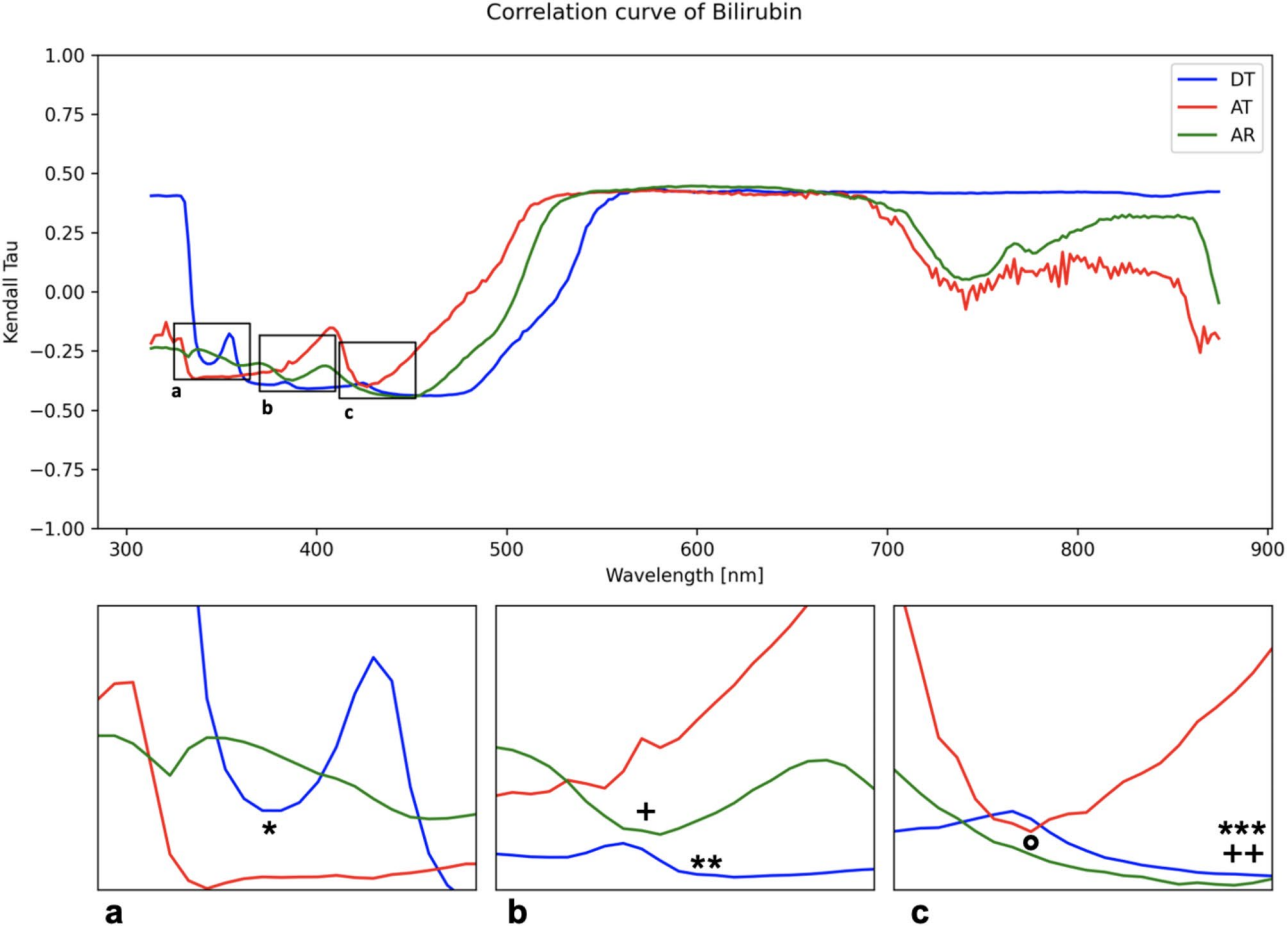
Correlation curve for the parameter bilirubin. The graph presents the correlation curves for the light pathways DT_20 (DT_EX1; blue), AT200 (AT_EX2; red), and AR320 (AR_EX2; green). The y-axis indicates the Kendall-Tau correlation coefficient, while the x-axis shows the wavelength in nanometers (nm). Subfigures (**a**, **b**, and **c**) provide enlarged views of the marked areas in the full spectrum. Symbols *(DT), +(AR) and °(AT) highlight the wavelengths selected for further statistical analysis.

As an example, Fig. 4 illustrates the correlation curves for all light pathways of the parameter bilirubin.

Figure 4 displays the correlation curves for light pathways DT_20 (DT_EX1; blue), AT200 (AT_EX2; red), and AR320 (AR_EX2; green). The Kendall-Tau correlation coefficient is represented on the y-axis, while the wavelength in nm is depicted on the x-axis. Based on this curve, wavelengths DT_20_344.37 nm (*), DT_20_387.38 nm (**), AR_320_387.38 m (+), DT_20_426.49 nm (***), AT_200_426.49 nm (°) and AR_320_426.49 nm (++) were identified as the most suitable for model generation in R and were thus selected as independent variables.

The selection of independent variables at wavelengths 344.37 nm and 387.37 nm was solely based on the correlation curves shown in Fig. 4. Variables at wavelength 426.49 nm were also chosen based on the correlation curve and literature research. Lee et al. reported a maximum of spectral absorption at wavelengths around 440 nm, which shifted to wavelengths of 415 nm to 420 nm in aqueous buffered solution, attributed to the oxidation of the pigment^22^.

The formation of a plateau in the correlation curves, starting at approximately 500 nm (see Fig. 4), was observed to varying degrees across most parameters. These plateaus were considered nonspecific, as preliminary statistical analyses demonstrated no benefit from including them. Therefore, wavelengths within these plateaus were excluded from further statistical analysis.

The same process was conducted for all other urine parameters.

No significant correlations were identified for both qualitatively and quantitatively measured glucose parameter as well as albumin. As a result, statistical analysis was terminated for glucose and albumin at this juncture.

All correlation coefficients guiding the selection of independent variables for further analysis were statistically significant, with a p-value of < 0.05.

### Statistical modeling

For the statistical modeling of urinary parameters, bilirubin was selected as an illustrative example. The final global models were developed in both the LR and LRRE models, utilizing the covariates AR_320_426.49 nm, DT_20_344.37 nm and DT_20_426.49 nm to classify bilirubin levels.

All variables, including the intercept, exhibited statistical significance with a p-value lower than 0.05. The final global models for all parameters can be found online in Supplementary Table S1.

The inner-sample performance of the LRRE model showed balanced accuracies (BACs) ranging from 0.731 to 0.889, with areas under the curve (AUCs) between 0.916 and 0.981. In comparison, the LR model achieved BACs between 0.721 and 0.777, with AUCs ranging from 0.915 to 0.945. These ranges reflect the performance across the five cross-validation (CV) models generated for each approach.

**Fig. 5:**
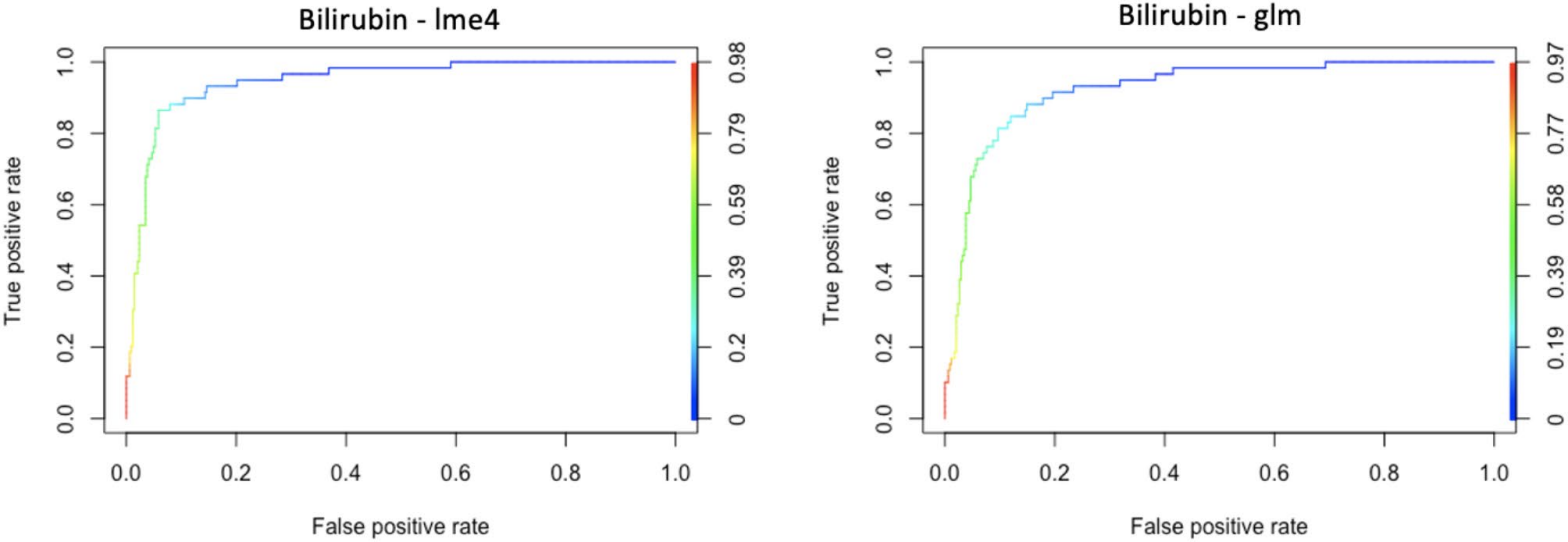
Area under the curve (AUC) analysis for LRRE (lme4) and LR (glm) models of bilirubin. The graph plots the false positive rate on the x-axis and the true positive rate on the left y-axis. The right y-axis displays the area under the curve.

For the global models, the LRRE model achieved a BAC of 0.771 and an AUC of 0.947, whereas the LR model attained a BAC of 0.735 and an AUC of 0.923. Refer to Fig. 5 for a visual representation of the ROC curves for both models.

Out-of-sample performance metrics for the LRRE model yielded BACs between 0.650 and 0.873 and AUCs ranging from 0.861 to 0.961. In comparison, the LR model exhibited BACs between 0.650 and 0.827 and AUCs between 0.866 and 0.959. Mean differences between inner and out-of-sample performance were computed for the LRRE model as 0.060 (BAC) and 0.026 (AUC). Consequently, the final corrected global LRRE model for Bilirubin yielded a BAC of 0.711 and an AUC of 0.921.

For the LR model, mean differences were calculated as 0.007 (BAC) and 0.002 (AUC). Thus, the final corrected global LR model for Bilirubin achieved a BAC and AUC of 0.728 and 0.921, respectively.

Table 2 summarizes the corrected AUC and BAC for each parameter and model, including the number of variables within the models.

**Table 2:** Comparative analysis of corrected area under the curve (AUC), corrected balanced accuracy (BAC), and number of variables in models for all parameters.

Urine marker	AUC (LRRE)	AUC (LR)	BAC (LRRE)	BAC (LR)	Covariates (LRRE)	Covariates (LR)
Bilirubin	0.921	0.921	0.711	0.728	3	3
Erythrocytes	0.814	0.771	0.709	0.655	4	4
Ketones	0.686	0.757	0.521	0.500	3	1
Leucocytes	0.764	0.659	0.703	0.571	3	3
Nitrite	0.549	0.681	0.527	0.536	2	2
pH acidic	0.814	0.787	0.699	0.674	6	6
pH basic	0.803	0.787	0.655	0.597	6	6
Protein	0.857	0.836	0.755	0.735	5	5
Specific gravity	0.851	0.851	0.599	0.575	6	3
Urobilinogen	0.871	0.843	0.707	0.637	2	4

Bilirubin demonstrates the highest AUC values in both LR and LRRE models. Similarly, parameters such as erythrocytes, pH parameters, protein, specific gravity, and urobilinogen exhibit favorable AUC and BAC values. Conversely, leukocytes, nitrite, and ketones display lower AUCs and BACs. The covariates columns denote the number of wavelengths incorporated into the final model, excluding the random intercept (1 | Patient) in the covariates (LRRE) column.

As depicted in Table 2, Bilirubin exhibited the highest AUC values in both logistic regression and logistic regression with random intercept models, along with high BAC values. Especially in the case of the AUC, no noteworthy difference can be observed between LRRE and LR.

Similarly, erythrocytes, pH parameters, protein, specific gravity, and urobilinogen demonstrated high AUC and BAC values. The inclusion of random effect intercepts correcting for multiple measurements in the same patient in these models generally led to classification improvements.

Parameter leukocytes, nitrite and ketones showcased lower AUCs and BACs. Although the inclusion of random effects improved the models for leukocytes, it had a varying impact on nitrite and ketones.

Quality interpretation of the models using the area under the curve (AUC) provides comprehensive information about the model’s performance across all possible separation values used for classification. BAC instead yields a classification measure that is easier to interpret.

The covariates columns in Table 2 indicate the number of wavelengths included in the final model. This count excludes the random intercept of the LRRE model, facilitating a better comparison of the number of wavelengths comprising the final LRRE and LR models.

Detailed information on the final models for each parameter, including mean differences and AUC plots, can be found online in Supplementary Table S2 and Supplementary Figure S4.

## Discussion

Despite significant advancements in digitalization, the analysis of urine samples from catheter bags remains largely analog. This traditional method is often inaccurate, incomplete, labor-intensive, and time-consuming, with results delayed by the inherent processes of charting and laboratory analysis. Here, we present a functional prototype of an intelligent sensor that utilizes a spectrometer to analyze urine output in real-time, continuously delivering results in a digital format. While this work represents an initial proof of concept, it demonstrates the feasibility and promise of such a system in addressing current challenges in urine analysis for catheterized patients.

The interpretation of AUC values, while sometimes contentious, generally follows the subsequent guidelines. Values below 0.6 indicate poor discrimination; values between 0.7 and 0.8 range from poor to moderate to good; values above 0.8 are typically considered good, and those exceeding 0.9 are very good^23^. Applying this framework, our models demonstrate the following discrimination abilities: both bilirubin models exhibit very good discrimination; at least one model for erythrocytes, acidic and basic pH, protein, specific gravity, and urobilinogen shows good discrimination. The leukocyte model with random effects and the ketone model without random effects demonstrate moderate discrimination, whereas both nitrite models exhibit poor discrimination.

Parameters with known absorption within the visible light spectrum, such as bilirubin, urobilinogen, and erythrocytes, were well-measured by the prototype, producing models with good to very good ROC curves and predictive capabilities. Similarly, multifactorially determined parameters like pH and specific gravity also generated models with good discriminative ability, suggesting that other components influencing these parameters, such as organic acids and bases, could be spectrometrically detected. The parameter glucose, which lacks distinct absorption features within the visible light spectrum, showed minimal correlation and poor discriminability, as expected.

The identification of specific wavelengths contributing to the detection of individual urine parameters revealed varying alignment with absorption maxima reported in the literature. Parameters such as bilirubin, erythrocytes/hemoglobin, and urobilinogen displayed strong concordance with previously characterized absorption maxima, suggesting robust detection capabilities. In contrast, parameters like ketones, leukocytes, and nitrite exhibited deviations from known absorption maxima, possibly influenced by factors such as pH variability, sample composition, or weak absorption properties. Proteins displayed a broader range of detected wavelengths, which may reflect the diverse molecular properties and interactions of protein subtypes in urine. Notably, parameters like pH and specific gravity, which lack defined absorption maxima, were measured based on broader spectral contributions rather than specific wavelengths. It should be emphasized that most reference values in the literature were obtained in water, whereas this study investigated biological urine samples, introducing additional complexity due to compositional variability. Overall, the results indicate that for markers with well-defined absorption properties, our approach reliably identified relevant wavelengths, even in the complex and variable matrix of urine. For markers with less distinct or poorly characterized absorption features, further optimization or alternative approaches may be required to improve detection accuracy. Detailed wavelength data and literature comparisons are provided in Table S3 in the supplementary material.

In catheterized scenarios, urine flow is typically slow and continuous, making significant compositional changes during measurement unlikely. Consequently, it was assumed that the real-life scenario could be approximated as a quasi-stationary state with respect to the flow velocity of the liquid. To approximate these real-life conditions, the sample was manually injected into the measurement chamber and measured at rest. As a result, the potential influence of liquid motion on the measurement process was not evaluated in this study. Future investigations conducted directly at the bedside will incorporate measurements of urine in flow to address this limitation.

Our findings demonstrate that urine particles absorbing within the measured light spectrum and multifactorially determined parameters like pH can be confidently predicted on a binary scale. Analysis of mean differences between inner and out-of-sample sample performance indicates lower values in logistic regression models without random effects compared to logistic regression models with random effects, suggesting a tendency toward overfitting in models including random effects. Nonetheless, after adjusting for overfitting, models with random effects generally outperform others.

The sample set used in this study was representative of the patient population in the Department of General, Visceral, and Transplantation Surgery, skewing toward healthy samples (Table 1). While this reflects real-world scenarios, it limits statistical analysis, particularly for parameters with fewer pathological samples. Larger, more balanced datasets in future studies could yield greater statistical power and improved performance estimates.

Current limitations in urine diagnostics from catheter bags include documentation errors and the time-consuming nature of manual processes. By enabling continuous measurement of urine contents, the proposed system could facilitate earlier complication detection, potentially improving patient outcomes. Our research group is also developing a system for integration into the Smart Catheter to accurately measure urine flow, which would further reduce nursing workload and enable precise fluid balance determination in catheterized patients.

Additionally, the data from this study are being analyzed using advanced AI methodologies by the Institute for Artificial Intelligence in Medicine (IKIM). Preliminary results from Partial Least Square Discriminant Analysis, random forests, and Convolutional Neural Networks confirm or improve upon our AUC and BAC results (to be published). Once the most effective AI approach is established, we aim to develop a digital fingerprinting system for early complication detection and hospital stay prediction.

The evaluation platform depicted in Fig. 1, though large, was designed to assess multiple light paths simultaneously, prioritizing functionality over compactness. The final product will incorporate only the most effective light path(s), allowing for optimization and miniaturization. We anticipate that the final optoelectronic setup will measure approximately 3 × 3 × 6 cm, creating a compact, wireless diagnostic unit attachable to urinary catheters at the bedside. Additional sensors for urine volume monitoring could further enhance utility, alleviating the need for manual monitoring and charting by healthcare workers.

In summary, this study demonstrates the feasibility of a real-time, spectrometry-based sensor for urine analysis, representing an important step toward digitalized, continuous monitoring in catheterized patients. While challenges remain, this work lays the foundation for future developments. With continued refinement and integration of AI and flow-monitoring systems, this technology has the potential to significantly enhance patient care and clinical efficiency.

## Electronic supplementary material

Below is the link to the electronic supplementary material.

Supplementary Material 1

## Data Availability

Data and Materials that support the findings of this study have been deposited to the correspondence, Prof. Dr. Michael Berger.
