# Supplementary material for: Continuous spectroscopic monitoring of urinary catheter output: advancements and clinical implications
Source: Sci Rep. 2025 Mar 12;15:8617. doi: 10.1038/s41598-025-92802-2 (PMC11903876; doi:10.1038/s41598-025-92802-2)
Supplement: Supplementary file 1 — Supplementary Material 1 [file 41598_2025_92802_MOESM1_ESM.docx]

**Supplementary Material**


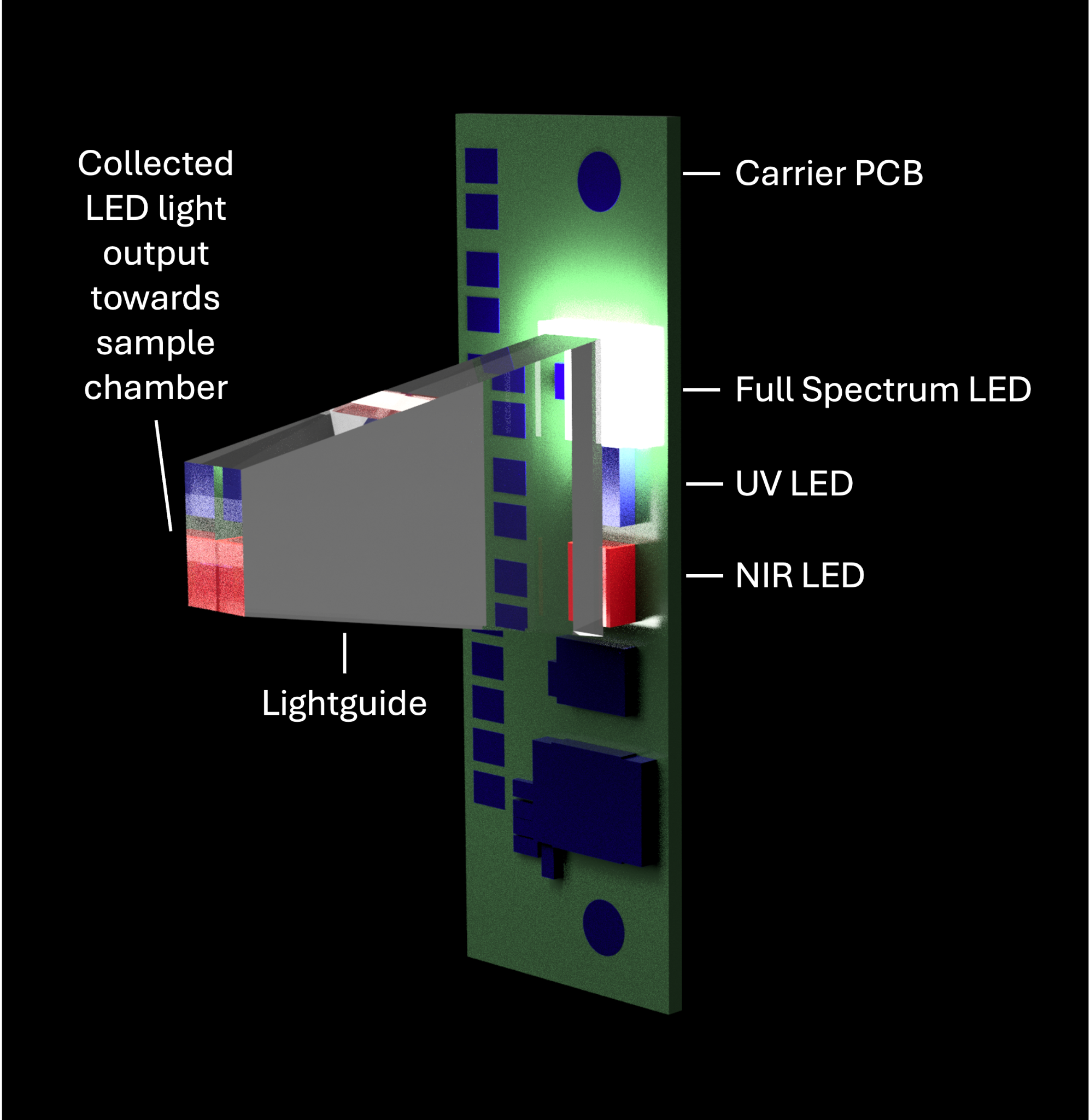


**Supplementary Figure S1:** Illustration of the hyperspectral light source. The emission spectrum of the three LEDs (NIR LED: Vishay VSMY3850, FS LED: Yuji P3210004.01, UV LED: Marktech MTSM340UV2-F512) were combined to construct a spectrum which covers the entire detection range of the mini spectrometer. The hyperspectral light is captured and directed to the sample chamber by the light guide. This figure was rendered using Autodesk Fusion 360.


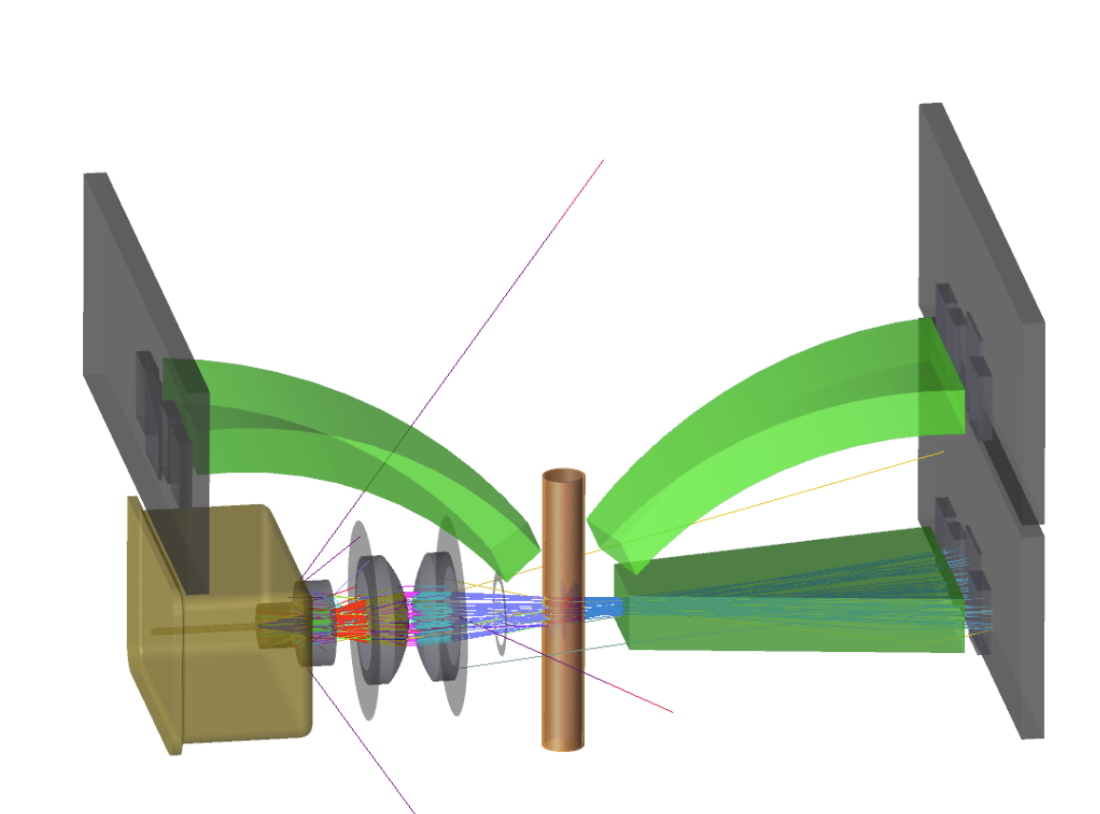


**Supplementary Figure S2:** Simulation of the different optical configurations to optimize optical performance using Zemax Optic Studio.


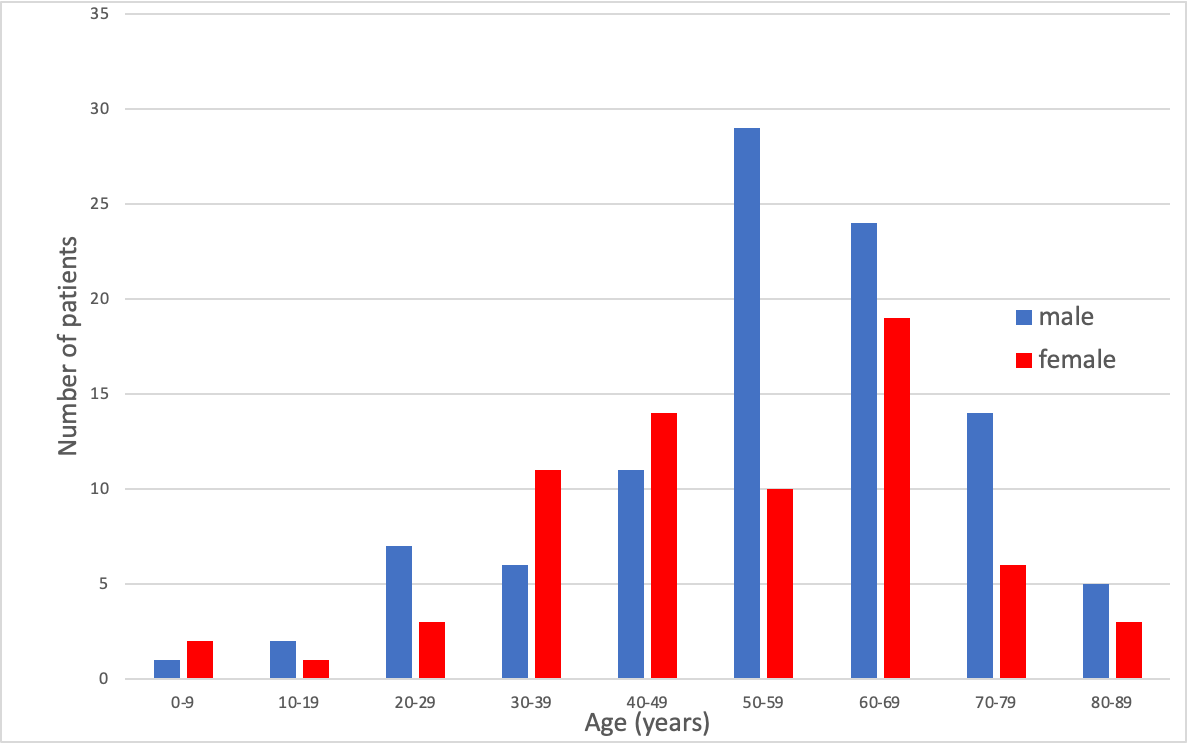


**Supplementary Figure S3:** Demographic distribution of the patient cohort. This figure illustrates the age distribution of the 168 patients, ranging from 0 to 85 years. The x-axis denotes age in 10-year increments, while the y-axis represents the number of patients. Each age group is further subdivided by gender, with red bars indicating female patients and blue bars indicating male patients.

**
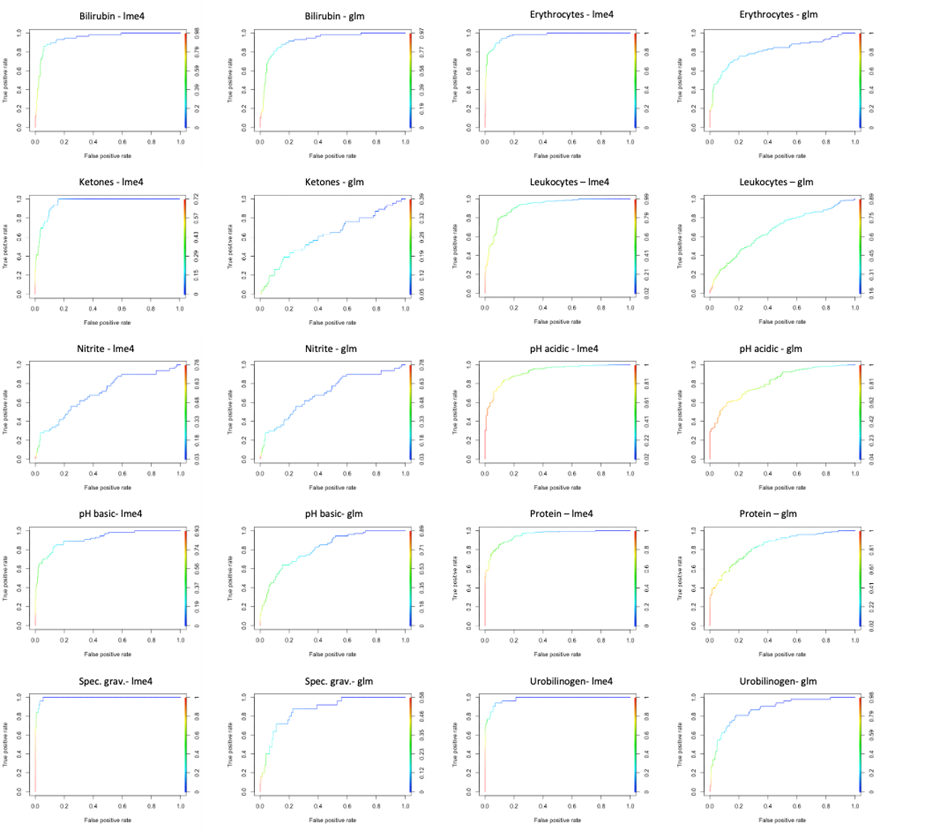
**

**Supplementary Figure S4:** Area under the curve (AUC) analysis for LRRE (lme4) and LR (glm) models of all parameters. The graph plots the false positive rate on the x-axis and the true positive rate on the left y-axis. The right y-axis displays the area under the curve.

**Supplementary Table S1:** Final models of urine parameters for logistic regression (LR) and logistic regression with random effects (LRRE).

| **Response** | **Model** | **Covariates** |
| --- | --- | --- |
| Bilirubin | LRRE | AR_320_426.49nm, DT_20_344.37nm, DT_20_426.49nm |
|  | LR | AR_320_426.49nm, DT_20_344.37nm, DT_20_426.49nm |
| Erythrocytes | LRRE | AR_320_416.71nm, AR_320_489.06nm, AR_320_573.14nm, DT_20_416.71nm |
|  | LR | AR_320_416.71nm, AR_320_489.06nm, AR_320_573.14nm, DT_20_416.71nm |
| Ketones | LRRE | AR_320_340.46nm, AR_320_506.66nm, AT_200_340.46nm |
|  | LR | AT_200_506.66nm |
| Leukocytes | LRRE | AR_320_344.37nm, DT_20_344.37nm, DT_20_395.21nm |
|  | LR | AR_320_344.37nm, DT_20_344.37nm, DT_20_395.21nm |
| Nitrite | LRRE | AT_200_440.18nm, DT_20_514.48nm |
|  | LR | AT_200_440.18nm, DT_20_514.48nm |
| pH acidic | LRRE | AR_320_571.19nm, AT_200_342.41nm, AT_200_430.4nm, DT_20_342.41nm, DT_20_449.96nm, DT_20_571.19nm |
|  | LR | AR_320_571.19nm, AT_200_342.41nm, AT_200_430.4nm, DT_20_342.41nm, DT_20_449.96nm, DT_20_571.19nm |
| pH basic | LRRE | AR_320_571.19nm, AT_200_342.41nm, AT_200_430.4nm, DT_20_342.41nm, DT_20_449.96nm, DT_20_571.19nm |
|  | LR | AR_320_571.19nm, AT_200_342.41nm, AT_200_430.4nm, DT_20_342.41nm, DT_20_449.96nm, DT_20_571.19nm |
| Protein | LRRE | AT_200_383.47nm, AT_200_516.44nm, DT_20_324.81nm, DT_20_387.38nm, DT_20_416.71nm |
|  | LR | Protein ~ AT_200_383.47nm + AT_200_516.44nm + DT_20_324.81nm + DT_20_387.38nm + DT_20_416.71nm |
| Spec. gravity | LRRE | AR_320_389.34nm, AR_320_571.19nm, AT_200_344.37nm, AT_200_426.49nm, AT_200_571.19nm, DT_20_344.37nm |
|  | LR | AR_320_389.34nm, AR_320_571.19nm, AT_200_344.37nm |
| Urobilinogen | LRRE | AR_320_383.47nm, AR_320_485.15nm |
|  | LR | AR_320_383.47nm, AR_320_432.36nm, AR_320_485.15nm, DT_20_485.15nm |

**Supplementary Table S2:** Mean differences of urine parameters for both logistic regression (LR) and logistic regression with random effects (LRRE).

| **Response** | **Model** | **Mean diff. BAC** | **Mean diff. AUC** |
| --- | --- | --- | --- |
| Bilirubin | LRRE | 0.060 | 0.026 |
|  | LR | 0.007 | 0.002 |
| Erythrocytes | LRRE | 0.135 | 0.157 |
|  | LR | 0.148 | 0.047 |
| Ketones | LRRE | 0.097 | 0.273 |
|  | LR | 0.015 | -0.138 |
| Leukocytes | LRRE | 0.143 | 0.159 |
|  | LR | 0.030 | 0.010 |
| Nitrite | LRRE | -0.003 | 0.151 |
|  | LR | -0.011 | 0.020 |
| pH acidic | LRRE | 0.110 | 0.109 |
|  | LR | 0.036 | 0.032 |
| pH basic | LRRE | 0.091 | 0.054 |
|  | LR | 0.009 | 0.029 |
| Protein | LRRE | 0.122 | 0.095 |
|  | LR | 0.016 | 0.018 |
| Spec. gravity | LRRE | 0.317 | 0.143 |
|  | LR | -0.037 | 0.020 |
| Urobilinogen | LRRE | 0.139 | 0.109 |
|  | LR | 0.020 | 0.031 |

**Supplementary Table S3:** Comparison of Literature-Reported Absorption Wavelengths with Observed Wavelengths for Urine Parameters.

| **Urine Parameter** | **Literature Wavelengths (nm)** | **Wavelengths observed in Current Study**  **(nm)** | **Notes** |
| --- | --- | --- | --- |
| **Bilirubin** | 440 (shifting to 425 in aqueous solutions, pH-dependent) [22] | 426, 345 | Good correspondence with literature, including pH-dependent shifts. |
| **Erythrocytes** | 380–420, 540, 576 [24] | 417, 489, 573 | Generally consistent with literature; slight discrepancy observed for 489 nm. |
| **Ketones** | UV-Region [25] | 340, 506 | Deviations noted; further investigation into ketone-specific absorptions in urine is required. |
| **Leucocytes** | No significant absorption described | 344, 395 | Observed absorption could suggest unique properties in urine samples. |
| **Nitrite** | 342 [26]; also reported UV-range  [27] | 414, 540 | Deviations noted; further investigation into nitrite-specific absorptions in urine is required. |
| **pH and Specific Gravity** | Not associated with specific absorption maxima | Diverse wavelengths; see supplementary table S4 | No characteristic maxima expected due to the nature of the parameters. |
| **Protein** | 205, 280 [28] | 325, 384, 387, 417, 516 | Observed wavelengths differ from standard protein absorptions; may reflect urine-specific effects. |
| **Urobilinogen** | 488 (oxidized to urobilin) [29] | 384, 432, 485 | Close match to literature for oxidized product; differences for related peaks. |
| **Glucose** | Infrared regions [30] | Not identified | No significant absorption observed; aligns with expectations given glucose absorption properties. |
